# Associations of Collectivism with Relationship Commitment, Passion, and Mate Preferences: Opposing Roles of Parental Influence and Family Allocentrism

**DOI:** 10.1371/journal.pone.0117374

**Published:** 2015-02-26

**Authors:** Kathrine Bejanyan, Tara C. Marshall, Nelli Ferenczi

**Affiliations:** Psychology Department, Brunel University, London, United Kingdom; University of Utah, UNITED STATES

## Abstract

In collectivist cultures, families tend to be characterized by respect for parental authority and strong, interdependent ties. Do these aspects of collectivism exert countervailing pressures on mate choices and relationship quality? In the present research, we found that collectivism was associated with greater acceptance of parental influence over mate choice, thereby driving relationship commitment down (Studies 1 and 2), but collectivism was also associated with stronger family ties (referred to as family allocentrism), which drove commitment up (Study 2). Along similar lines, Study 1 found that collectivists’ greater acceptance of parental influence on mate choice contributed to their reduced relationship passion, whereas Study 2 found that their greater family allocentrism may have enhanced their passion. Study 2 also revealed that collectivists may have reported a smaller discrepancy between their own preferences for mates high in warmth and trustworthiness and their perception of their parents’ preferences for these qualities because of their stronger family allocentrism. However, their higher tolerance of parental influence may have also contributed to a smaller discrepancy in their mate preferences versus their perceptions of their parents’ preferences for qualities signifying status and resources. Implications for the roles of collectivism, parental influence, and family allocentrism in relationship quality and mate selection will be discussed.

## Introduction

Parents have traditionally played a large part in their children’s mate selection [1], exerting influence by approving or even choosing their children’s marital partner for them. Western cultures, with their emphasis on personal desires and independence [2], have long since moved away from this practice; individuals are expected to exercise personal control over their own partner selection and relationship maintenance. Conversely, a higher degree of parental influence on mate choice and relationship functioning is still evident among Eastern, collectivistic cultures, where greater emphasis is placed on family cohesion and the needs of the group over those of the individual [3]. As a consequence of parental influence, individuals sometimes date in secrecy, exercising temporary liberties over their own partner choice until they are expected to abide by parental expectations and choose a marital partner congruent with their parents’ standards [4]. Accordingly, while individuals try to reconcile their personal needs with those of familial and cultural expectations, the degree of passion and commitment they feel towards their romantic partner may change.

While parental influence highlights the authority parents can have over their children’s choices, family allocentrism—defined as the strength of closeness and devotion between family members [5]—can potentially influence the willingness for children to take their parents’ opinions into consideration when selecting a mate. Consequently, an equally important variable that may influence relationship quality and mate preferences—albeit less studied than parental influence—is the cultural value of family allocentrism. The closeness generated by family allocentrism may also set a high standard for desired levels of commitment and passion in children’s subsequent romantic relationships. Insofar as more collectivist cultures emphasize both family allocentrism and parental influence on mate choice, individuals from these cultures may feel opposing pressures on their romantic relationships: family allocentrism may drive commitment and passion up, while parental influence on mate choice may drive them down.

We chose to examine cultural influences on passion and commitment because they are two indices of relationship quality that are universally experienced, but heavily regulated by social norms across cultures. Passion has a strong sexual component [6], and from an evolutionary standpoint, all humans have the same capacity for sexual passion because of their biological propensity to reproduce [7]. However, while the inner experience of passion may be universal, the *expression* of passion in relationships may be prone to cultural variability, with certain cultures viewing it as a disruption to family dynamics and culturally-sanctioned marital arrangements [8, 9]. Relationship commitment, similar to passion, has some universal resonance. According to evolutionary psychologists, men need to demonstrate relationship commitment to gain sexual access to women; for women, securing commitment in a relationship is similarly valuable because it allows access to resources and ensures the long-term survival of offspring [10]. The importance of commitment across cultures is evident in studies showing that it is predictive of relationship growth and longevity across cultures [11, 12]. Still, cultures vary in the extent to which they value relationship commitment [13].

Furthermore, we were interested in investigating the difference between children’s mate preferences and what they perceive to be their parents’ mate preferences. Across cultures and historical eras, parents have made overt attempts to influence children’s mating behavior, such as arranging their marriages, or more subtle attempts at influence, such as bribing, regulating children’s social environment, or facilitating children’s interactions with others [14]. While parental influence over children’s mating choices has considerably decreased over the past few centuries, it nevertheless still exists in many cultures. In the following studies, we were interested in examining how different forms of parental involvement (i.e., parental influence on mate choice and family allocentrism) could potentially sway children to prefer partners who are aligned with parental expectations. Overall, the current studies examined whether parental influence and family allocentrism mediated the association of collectivism with commitment, passion, and parent-child discrepancies in preferences for marital partners. We begin with an overview of romantic relationships across cultures.

### Romantic Relationships in Individualistic and Collectivistic Cultures

While mate selection is universal, the process by which partners are selected and relationships are maintained is often determined by cultural and social factors. Individualistic cultures value self-sufficiency and the development of personal identity [1] and the foundation for relationships is built on the idealization of romantic love—a self-seeking, intrinsic desire [15]. Passion is frequently touted as the essence of love—the basis upon which love is cultivated [16].

Within this cultural milieu, a standard developmental trajectory in adolescence and early adulthood is to begin the progression towards individuation and to freely undertake the process of mate selection [17]. This is accomplished by becoming more autonomous from one’s parents and commencing the exploration of relationships with different partners to gain experience in the realm of love and sexuality [18]. Adolescents and parents work through this process, resolving and integrating the adolescent’s new identity as a young adult [19]. The parents’ authority and influence gradually reduce as adolescents take full responsibility for their own actions and decision-making processes.

In contrast, many Eastern cultures emphasize the values of group harmony and cohesion [3]. Individuals are socialized to consider the well-being of the group over that of their own personal needs. Customarily, mate selection has not been an enterprise an individual embarks on alone, but one in which the family plays an important role in assessing and, in some cases, ultimately selecting the individual’s lifetime partner for them [17]. Dating and engaging in sexual activity prior to marriage are often considered inappropriate in Eastern, collectivistic cultures [20, 21]. In these cultures, marriage is the acceptable venue for intimate contact and sexual relations with a romantic partner [22]. Therefore, collectivist parents often do not condone Western-style dating practices or spending time with opposite sex peers because it contradicts many traditional cultural practices regarding mate selection [4, 23]. Instead of placing emphasis on the romantic connection between individuals, parents encourage children to assign more weight to pragmatic qualities in a prospective partner such as economic resources, social and religious status and, often most importantly, positive interactions between the two families [23].

While cultural values fundamentally construe one’s worldview, in order to ensure that these ideals are sustained over time, parents need to play an active role in promoting and transmitting these beliefs from one generation to another. Within immigrant families, family allocentrism, rather than interdependence, tends to be a more effective means of transmitting preferences for traditional mates between parents and children [24, 25]. To further this area of research, the present studies examined the collectivist values of parental influence on mate choice and family allocentrism as predictors of parent-child discrepancies in marital preferences and two indices of relationship quality—commitment and passion.

### Predictors of Commitment

In Western, individualistic cultures, feelings of affection, tenderness and commitment are seen as necessary predecessors to marriage [26]. In many collectivist cultures, on the other hand, intimacy, closeness, love, commitment and sexual engagement are frequently intended to develop once the couple is married—not before [27]. Therefore, the phase of dating as an exploratory process during which young couples first cultivate feelings for one another and then determine if they are suited for marriage can contradict many traditional Eastern, collectivistic mate selection practices [28, 29].

With globalization, there is a growing trend in more collectivistic cultures for young adults to exercise greater personal choice in their mate selection and engage in dating [3] in spite of parents’ disapproval [30]. As a way to combat parental disapproval, young adults often conceal their romantic life from family and date without parental knowledge and consent. In their study of dating and sexual activity among Asian Americans, Lau, Markham, Flores, and Chacko [20] found that 70% of Asian-Americans dated in secrecy from their parents. In keeping with these findings, Manohar [21] found that Indian-American adolescents reported having to go to great lengths to hide their dating life from their family members. Insofar as premarital connections are treated very carefully in collectivistic cultures, partners commonly date in secret until they are ready to get married, and can then reveal their intentions to their families [4, 29]. However, the decision between the partners does not ensure the prospect of marriage, as both families also have to agree. Therefore, the commitment partners feel in these relationships can often waver, depending on the perceived acceptance of other family members. Macdonald, Marshall, Gere, Shimotomai and Lies [31] found that in collectivistic cultures, confidence in the relationship was derived at least in part from the approval of family members towards the couple’s relationship.

Accordingly, while a number of researchers have noted the importance of commitment to a romantic relationship for many collectivist couples [29, 32], this may primarily be the case for married couples and not necessarily apply to those in dating or premarital relationships. Instead, in premarital relationships, parental influence may drive down commitment because the union is not yet recognized as legitimate and integrated into family ties. Consequently, we expected individuals from collectivistic backgrounds to experience higher parental influence on their mate choice, and in turn, report feeling less committed to their romantic partner.

On the other hand, whilst collectivists’ tendency to accept high parental influence on mate choice may undermine commitment in premarital relationships, their family allocentrism may also bolster feelings of commitment to close others. In an attempt to preserve the heritage cultural value system and maintain solidarity within the familial unit, parents may strive to cultivate family allocentrism [33, 34]. Experiencing stronger family relationships—i.e., greater family allocentrism—allows for more efficient communication and transfer of heritage culture values from one generation of immigrants to successive generations [5]. Lay and colleagues [5] discovered that within their sample of Western participants who possessed some degree of ethnic identity, those who were higher in family allocentrism also showed greater adherence to their heritage cultural customs and group membership. By extension, Marshall [35] found that Chinese Canadians who identified more strongly with their collectivistic heritage culture also reported feeling more committed to their romantic relationships. A stronger connection to the family unit may lead to a stronger identification with heritage cultural values of commitment to in-group members. Therefore, in the following studies we expected to find that collectivists would report stronger family allocentrism, resulting in stronger commitment towards their romantic partner.

### Predictors of Passion

Western notions of romance and passion are increasingly influencing collectivists’ perceptions of romantic relationships [9]. For instance, the salience of Western romantic novels has increased in India, with many Indian women enthralled with these stories of passion and desire [36, 37]. Likewise, Bollywood movies routinely depict passionate interludes between lovers as they struggle against confining social norms and family obligations [32]. Moreover, Indian epics and mythology often venerate romantic love and passion between couples, while some Indian philosophers praise romantic love as the highest possible ideal individuals can reach [38, 39].

Likewise, many Chinese artworks and historical stories are permeated with tales of longing, passionate love and sexual desire [40]. Accordingly, many researchers have argued that passionate love may be a cross-cultural phenomenon and not just confined to the West [16, 41, 42]. In their study of adolescent romantic experiences, Regan and colleagues [18] found that 85% of the adolescents in their sample, regardless of cultural background, had experienced love.

While this may be the case, in many Eastern, collectivistic cultures, passionate love is seldom encouraged outside of movies and stories [43]. The expression of passionate love is often viewed not only as lewd and inappropriate, but many elders believe it poses a threat to family hierarchy by drawing children’s focus away from the family and onto the romantic partner [8, 44]. Under the thrall of passion, parents fear, children may gain the fortitude to act against their normative familial and cultural obligations, potentially jeopardizing the honor of the entire family [9]. Therefore, children may experience excessive pressure from parents to act pragmatically and suppress any feelings of passion for a romantic partner [45, 46]. This especially applies to the dating or courting period when parents may feel their children’s actions can pose a greater liability to the family; given that the romantic union has not yet been legitimatized through marriage, the risk of children disobeying parents or tarnishing the family’s reputation is particularly high [47]. Therefore, to the extent that collectivists accept greater parental influence on their mate selection, we predicted that they would report decreased feelings of passion within their relationship.

In addition to complying with parental wishes about who to marry and how to behave in a romantic relationship, collectivist youth may themselves try to actively restrain feelings of passion towards their romantic partner. They may do this not just because these feelings are often viewed as inappropriate in their cultural milieu, but also because they do not want to upset family ties. For instance, 60% of the Hindu men Derne [48] interviewed believed that partners should not spend too much time together because, in cultivating a closer, more romantic bond, they will inevitably neglect their parents and cultural duties. Therefore, parental influence and family allocentrism may work in tandem together to dampen down passion; parents try to influence their children’s romantic relationship to ensure focus still remains on the family, while children, in feeling close to family, do the same to ensure ongoing family harmony.

Alternatively, to the extent that family allocentrism creates close bonds between family members, this connection may be similarly desired within a romantic relationship. As a result, the feelings one develops towards a romantic partner can often be inextricably tied to the connection one feels towards family members. For instance, Indian participants reported that the greatest joy one can experience in a relationship is through nurturing stronger ties to family and religion [32]. Therefore, family allocentrism can elevate the value of a romantic relationship, idealizing it as a means of increasing overall familial happiness and closeness, thereby heightening feelings of passion and romance towards a partner.

Additionally, experiencing close family ties may create the desire to have a similar connection with a romantic partner. Derne [48] reported that in Hindu Indian families, the relationship between a younger son and his older sister-in-law can serve as an important channel through which a young man learns about romantic relationships. This relationship, which can frequently be sexually provocative and playful, but never acted upon by either party, can often cultivate the desire for a similar relationship with a marital partner. Thus, given these competing predictions for the association of family allocentrism with passion, we investigated this association on an exploratory basis only.

### Predictors of Parent-Child Discrepancies in Mate Choice

Romantic relationships exist cross-culturally, with parents and children often viewing marriage as the touchstone of a successful romantic union [1, 23]. Through this union, new bonds are established and families expand to incorporate in-laws and children. However, while both parents and children are affected by this match, the motivation behind forming a marital relationship may differ for each group [49, 50]. In collectivistic cultures, traditional family values are particularly central to group cohesion, with the institution of marriage playing a prominent role in the transference of these values [51]. Parents often utilize their children’s marital union as an important means for forging new alliances, strengthening social standing, and ensuring the continuity of family lineage [52].

For children, however, the partnership cultivated through marriage can help satisfy personal needs for emotional connection and fulfillment. As postulated by parent-offspring conflict theory over mate choice [14], the differing attitudes parents and children hold regarding the purpose of marriage may lead them to value different ideals in a marital partner. However, in cases where parents exert strong influence over their children’s mate choice, there may be an inclination for children to succumb to their parents’ wishes, especially in collectivistic societies where deferring to elders is both expected and commonplace. For instance, it has been reasoned that for collectivists, marital unions may not be the fulfillment of personal desires, but an outcome of family obligations [26]. Several studies have found that in the West marital happiness is motivated by factors that benefit the self, while factors that benefit social relationships are frequently associated with marital happiness in the East [8, 53]. Consequently, insofar as collectivists report higher parental influence on their mate choice, we expected them to report a smaller discrepancy between their choices for a marital partner versus their parents’ perceived choices.

Family allocentrism, on the other hand, strengthens bonds between family members, and may actually lead children to develop similar mate preferences to their parents’ preferences. Lalonde and colleagues [24] found that second-generation South Asian Canadians who identified more strongly with their heritage culture reported higher family allocentrism and, in turn, more traditional mate characteristics. Thus, stronger ties with family members may have transmitted the heritage culture value placed on traditional characteristics such as conventional gender role behavior in a potential spouse. In an environment where parents and children feel warmth and intimacy towards one another, they are also more likely to share related values and beliefs, thereby exhibiting similar preferences for a marital partner. A significant relational dynamic between emotional closeness of family members and willingness to accept parental messages is established—the closer children feel they are to their parents, the greater acceptance they have of parental values [54]. Therefore, we hypothesized that individuals from collectivistic backgrounds would experience higher family allocentrism and, in turn, report a smaller gap between their choices for a marital partner versus their parents’ perceived choices.

### The Present Research

Many studies have explored cultural influences on relationship quality and mate preferences [24, 55, 56, 57], but have primarily focused on the value assigned to specific mate attributes within an individualistic-collectivistic cultural milieu. Moreover, these studies have taken a Western standpoint by examining relationship quality from the perspective of the couple, not giving enough weight to the role of family involvement in this dynamic [3, 4]. To the extent that family influences collectivists’ marital partner preferences and relationship outcomes, the present studies fill a gap in the existing research by testing two facets of collectivism—parental influence and family allocentrism—as countervailing forces on romantic relationship outcomes. We propose that while parental influence and family allocentrism are both tenets of collectivism and positively associated with one another, they are separate constructs that can exert differential influences on mate preferences and relationship quality. While we expected that parental influence would drive collectivists’ commitment and passion down, we also expected family allocentrism to drive them up (Hypotheses 1 and 2, respectively). Meanwhile, we predicted that parental influence and family allocentrism would narrow the gap between collectivists’ marital preferences and their perceptions of their parents’ preferences (Hypothesis 3). Study 1 tested our hypotheses within a British sample, and classified participants as high or low in collectivism based on their heritage culture. Study 2 aimed to extend the findings of Study 1 by recruiting participants from two nations widely regarded as typifying high versus low collectivism—India and the United States.

## Study 1

### Ethics Statement

The Brunel University Psychology Research Ethics Committee provided ethical approval for Studies 1 and 2. Participants gave written informed consent at the beginning of the survey. All responses were confidential and anonymous.

### Participants

The sample consisted of 154 participants who currently resided in the United Kingdom (121 women and 33 men; *M*_age:_ 20.77, *SD*: 4.75). They were recruited through an advertisement placed on the authors’ university intranet site; if they completed the survey, they were given the option of entering a raffle to win a £20 gift certificate at a local shopping mall. Participants were further enlisted through the university’s undergraduate psychology participant pool; they were awarded one course credit upon completion of the survey. Prior to Study 1 data collection, power analyses were conducted using the software package called G* Power [58]. The analysis was two-tailed, consisted of an effect size of. 5, alpha level of *p* <. 05, power of. 8, and allocation ratio of 1. Results indicated that a sample size of 128 participants would be appropriate for our study. Of our total sample of 154 participants, 54% indicated they were currently single and the remaining 46% stated they were in a relationship (dating, cohabitating, engaged, or married). Because only participants involved in a relationship completed measures of commitment and passion, we have reported the demographic information separately for single and involved participants. The demographic information is presented in Table 1.

**Table 1:** Demographic Statistics for Study 1.

Demographic Variables	Single Participants	Participants in a Relationship
	%	%
GENDER:		
Male	25	83
Female	75	17
AGE:	*M* = 20.23	*M* = 21.41
	*SD* = 3.89	*SD* = 5.56
DATING STATUS:		
Date regularly	70	89
Do not date regularly	30	11
PARENTS KNOW ABOUT RELATIONSHIP:		
Yes		78
No		22
UK BORN:		
Yes	76	69
No	24	31
*LENGTH IN UK:	*M* = 17.81	*M* = 69.69
	*SD* = 40.97	*SD* = 66.87
ETHNICITY:		
White/Caucasian	34	63
Afro-Caribbean	18	9
Asian	30	7
South East Asian	2	3
Mixed	11	4
Other	5	14
HERITAGE CULTURE:		
British	33	47
European	5	18
South Asian	27	11
Middle Eastern	2	6
African	18	7
Caribbean	8	4
Southeast/East Asian	6	3
North/Latin American	1	4
PARTNER’S ETHNICITY:		
White/Caucasian		68
Afro-Caribbean		4
Asian		7
South Asian		3
Mixed		4
Other		14

* Length of time in months participants have lived in the UK who were not born there.

### Procedure and Materials

An online survey was generated through a survey-development website (www.surveymonkey.com). Participants were initially presented with demographic questions, and at the end of this section, they were asked to indicate their current relationship status. Individuals who indicated that they were currently involved in a romantic relationship were directed to complete a section on the level of commitment and passion they felt in their relationship before moving on to the other measures.

#### Collectivism

Participants were asked to indicate their heritage culture. In line with Hofstede’s [59] ratings of nation-level collectivism, we created an effect-coded variable that distinguished between participants from more collectivistic heritage cultures (1 = South Asian, African, Middle Eastern, Latin American, East Asian, Southeast Asian) and less collectivistic ones (-1 = British, European, Caribbean, North American). To test whether those who were categorized as high or low in collectivism did, in fact, differ in their degree of collectivism we also administered the collectivism subscale of the Horizontal/Vertical Individualism/Collectivism Scale [60]. The collectivism scale consists of 8 items; sample items include, “My happiness depends very much on the happiness of those around me” and “I usually sacrifice my self-interest for the benefit of my group”. Responses were measured on a 5-point Likert scale ranging from 1 *(Strongly disagree)* to 5 *(Strongly agree)*. We collapsed across the horizontal-vertical dimension to increase the reliability of the scale. Cronbach’s alpha was. 72.

#### Parental Influence on Mate Choice

The 10-item Parental Influence on Mate Choice Scale by Buunk, Park and Duncan [3] assesses acceptance of parental involvement in children’s mate choice. This scale was created with the intention that it could be utilized within diverse cultural groups. Example items include “It is the duty of parents to find the right partner for their children, and it is the duty of children to accept the choice of their parents” and “If their parents have serious objections against someone their children prefer as a partner, children should break off the relationship with that person.” Responses were measured with a 5-point Likert scale (1 = *Strongly disagree*, 5 = *Strongly agree*). Cronbach’s alpha was. 77.

#### Family Allocentrism

The Family Allocentrism Scale [5] is comprised of 21 items. This scale measures the extent of closeness a person feels towards his or her family. Example items are “The opinions of my family are important to me” and “My happiness depends on the happiness of my family”. Participants used a 5-point Likert scale (1 *= Strongly disagree*, 5 *= Strongly agree*) to indicate their level of agreement with each item. Cronbach’s alpha was. 80.

#### Preferred Mate Attributes

Eighteen items were taken from the Preferred Mate Attributes Scale [55] to measure the desirability of a range of mate characteristics (e.g., sociability, similar education, desire for home and children). Participants rated the importance of each attribute for a potential marital partner. Next, they were asked to reflect on their parents’ point of view and rate each item according to how important it would be to their parents for the participant’s potential marital partner to possess the characteristic. Ratings were made on a 4-point Likert scale *(0 = Irrelevant/Unimportant*, *3 = Indispensible/Very Important)*. Cronbach’s alpha was. 82 for participants’ own marital partner preferences and. 84 for participants’ perception of parental mate preferences. Principal components analysis indicated that a single dominant factor accounted for 28% and 29% of the total variance in one’s own and perceptions of parents’ mate preferences, respectively; additional factors accounting for small portions of the total variance were not interpretable. To assess the discrepancy between the participant’s preferences for a marital partner and perceived parental preferences for the participant’s marital partner, we calculated the absolute difference between total scores for marital preferences and parental preferences. This new score was utilized in the analyses to indicate parent-child discrepancy in mate preferences, with larger scores representing larger discrepancies.

#### Commitment

Seven items from the Investment Model Scale [61] were used to measure commitment for participants currently involved in a romantic relationship. Examples of the items include “I am committed to maintaining my relationship with my partner” and “I feel very attached to our relationship—very strongly linked to my partner”. The items were measured with a 5-point Likert scale (1 *= Strongly disagree*, 5 *= Strongly agree*) and were highly reliable (α = .94).

#### Passion

Fifteen items taken from the Triangular Love Scale [62] were used to assess passion (e.g., “Just seeing my partner is exciting for me” and “I adore my partner”) for those individuals who indicated they were currently involved in a romantic relationship. A 7-point Likert scale was used ranging from 1(*Not at all*) to 7 (*Extremely*). Internal consistency was high (α = .95).

#### Demographic questions

Participants stated their sex, age, where they were born, and where they currently resided.

### Results and Discussion

Means, standard deviations, and Pearson’s correlations are reported in Table 2. We created an effect-coded variable to differentiate between participants born in the UK (1) from those who were not (-1). Additionally, to ascertain whether collectivism, family allocentrism, and parental influence contributed to participants’ mate preferences over and above whether the participant had any previous experience in choosing a mate, we constructed an effect-coded variable to distinguish participants who had regularly dated (1) from those who had not (-1). Sex (1 = male, -1 = female) and age were also included as predictors in the model. We also included parents’ awareness of their children’s relationship as a control variable in our analysis. However, the results of our analyses were not influenced by this variable, and therefore it was removed from our subsequent mediational models. Finally, we conducted a *t*-test to see whether participants categorized as high in collectivism according to Hofstede’s [59] ratings of nation-level collectivism were indeed more collectivistic than those participants categorized as low in collectivism. The results of the analysis showed that participants who were categorized as high in collectivism (*M* = 29.02, *SE* = 4.94) did in fact score higher in collectivism compared to those who were categorized as low in collectivism (*M* = 27.21, *SE* = 3.99), *t*(151) = -2.41 *p* <. 02. For participants who were designated as low in collectivism, 81% were born in the UK, while 19% were not; from those high in collectivism, 38% were born in the UK, while 62% were born elsewhere. We decided to use the categorical measure of collectivism in Studies 1 and 2 for greater clarity of interpretation in our analyses.

**Table 2:** Pearson’s correlations and descriptive statistics for Study 1.

Variable	1	2	3	4	5	6	7	8	9	10	Mean	*SD*
1. Sex	1.00										-	-
2. Age	.21**	1.00									20.77	4.75
3. Regularly Date	.00	.10	1.00								-	-
4. UK Born	.00	-.16^†^	.00	1.00							-	-
5. Collectivism	.03	-.09	-.26**	-.22**	1.00						-	-
6. Family Allocentrism	-.14	-.15^†^	-.12	-.15^†^	.15^†^	1.00					68.56	10.66
7. Parental Influence	.01	-.11	-.32**	-.22**	.50**	.40**	1.00				18.48	5.96
8. Commitment	.05	.22^†^	-.17	-.10	.22^†^	.12	-.10	1.00			28.54	7.39
9. Passion	.15	.15	-.03	-.10	.14	.05	-.19	.82**	1.00		79.03	20.02
10. Parent-Child Discrepancy in Mate Preferences	.11	.11	.21^†^	-.04	.08	.25**	-.10	.21	.22^†^	1.00	4.73	4.48

^†^ *p* <. 10.

*p <. 05.

**p <. 01

**Fig 1:**
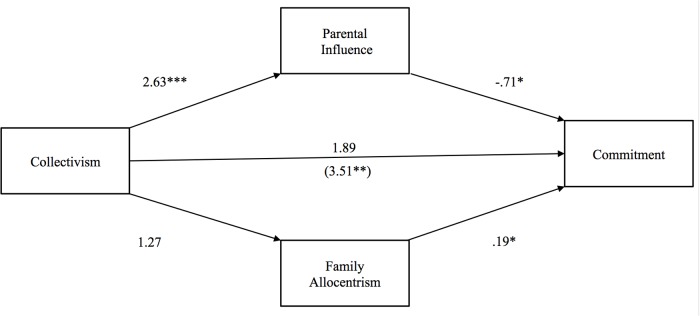
Study 1: Indirect effect of collectivism on relationship commitment through parental influence and family allocentrism. The value in parentheses represents the direct effect, and the value directly above is the total effect. * p <. 05, ** p <. 01, ***p <. 001.

**Fig 2:**
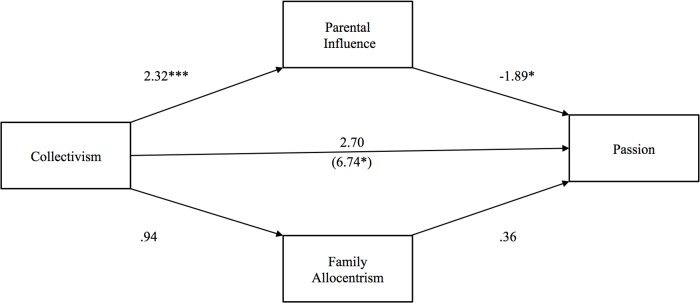
Study 1: Indirect effect of collectivism on relationship passion through parental influence and family allocentrism. The value in parentheses represents the direct effect, and the value directly above is the total effect. * p <. 05, ** p <. 01, ***p <. 001.

**Fig 3:**
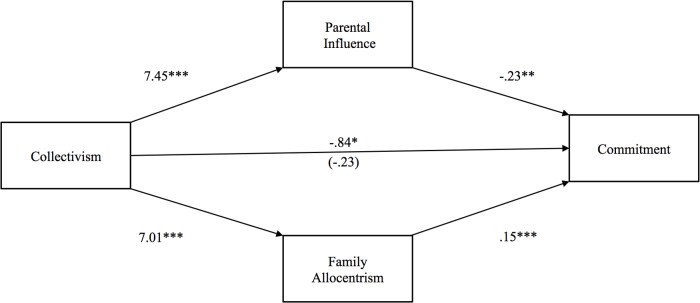
Study 2: Indirect effect of collectivism on relationship commitment through parental influence and family allocentrism. The value in parentheses represents the direct effect, and the value directly above is the total effect. * p <. 05, ** p <. 01, ***p <. 001.

**Fig 4:**
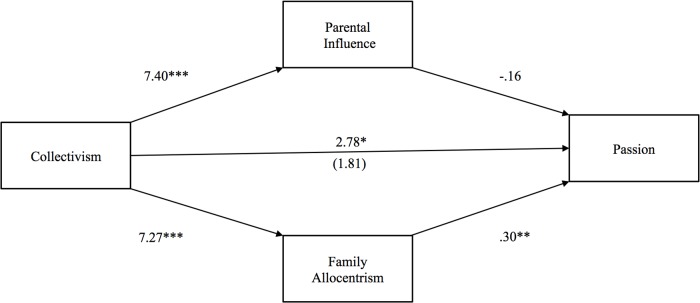
Study 2: Indirect effect of collectivism on relationship passion through parental influence and family allocentrism. The value in parentheses represents the direct effect, and the value directly above is the total effect. * p <. 05, ** p <. 01, ***p <. 001.

**Fig 5:**
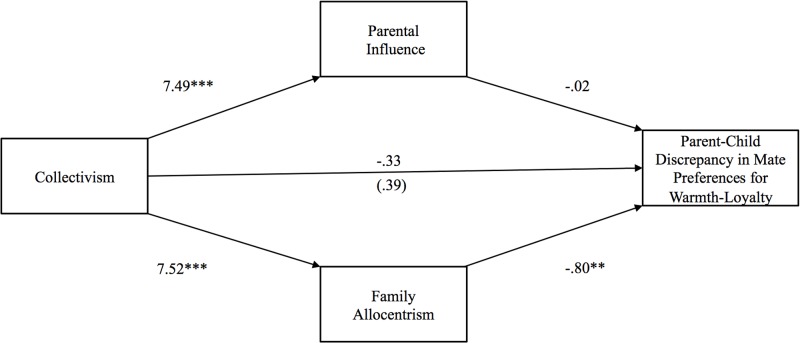
Study 2: Indirect effect of collectivism on parent-child discrepancy in mate selection for qualities signifying warmth-loyalty through parental influence and family allocentrism. The value in parentheses represents the direct effect, and the value directly above is the total effect. * p <. 05, ** p <. 01, ***p <. 001.

For our mediational models, we assessed the association of collectivism with commitment via parental influence and family allocentrism with Preacher and Hayes’s [63] SPSS script for testing multiple mediation effects. Their bootstrap method tests a purported causal sequence in which an independent variable exerts an indirect effect on a dependent variable through a mediating variable [64]. When mediation occurs, the *total effect*—which does not control for the mediating variable—should be larger than the *direct effect*, which controls for the mediating variable. In the case of suppression, the inclusion of a mediator results in a direct effect that is *larger* than the total effect [65], thereby strengthening the relationship between the independent and dependent variables. In the following models, then, collectivism was the independent variable, parental influence and family allocentrism were the mediators, and commitment, passion, and parent-child discrepancy were the respective dependent variables. As seen in Figs. 1–5, the regression coefficients for collectivism predicting parental influence and family allocentrism varied slightly within each study depending on the dependent variable. This was due to missing data.

The first model tested Hypothesis 1—that collectivism would exert a negative indirect effect on commitment through parental influence, but a positive indirect effect through family allocentrism. As seen in Fig. 1, the direct effect of collectivism on commitment (i.e., controlling for parental influence and family allocentrism) was larger and significant (*b* = 3.51, *p* <. 01) compared to the total effect (*b* = 1.89, *p* >. 05). When the independent variable is dichotomous, Preacher and Hayes [63] recommend reporting unstandardized regression coefficients. Examination of the 95% bias-corrected confidence intervals (CI) from 1,000 bootstrap samples revealed that the indirect effect of collectivism on commitment through parental influence was negative and significant [*b* = -1.90 (CI: -3.97, -.34)], partially confirming our hypothesis. The indirect effect through family allocentrism was not significant.

The second hypothesis tested whether collectivism would exert a negative indirect effect on passion through parental influence, but a positive indirect effect through family allocentrism. Paralleling the results for commitment, the direct effect of collectivism (*b* = 6.74, *p* <. 05) on passion was significant and larger than the total effect (*b* = 2.70, *p* >. 05), partly confirming our second hypothesis (see Fig. 2). The indirect effect of collectivism on passion through parental influence was negative and significant [*b* = -4.42 (CI: -9.88, -.90)], whereas the indirect effect of family allocentrism was not. Disconfirming our third hypothesis, neither parental influence nor family allocentrism mediated the relationship between collectivism and parent-child discrepancy in mate preferences.

Overall, these results partially corroborated our predictions: a positive association of collectivism with commitment and passion was counteracted by collectivists’ tendency to experience higher parental influence on their romantic relationships. When parental influence was not accounted for in the model, there was no significant association of collectivism with commitment or passion; when parental influence was controlled, a positive association of collectivism with commitment and passion emerged. Contrary to our predictions, family allocentrism did not account for these positive associations. That collectivism was positively but non-significantly associated with family allocentrism may have been responsible for our inability to support this hypothesis. We sought to confirm the association of collectivism with family allocentrism in Study 2 by sampling from two cultures that more clearly differed in collectivism.

## Study 2

The aim of Study 2 was to replicate and extend the results of Study 1 in three key ways. First, we collected data from two cultures that more clearly differed in collectivism. A limitation of Study 1 was that all participants were residents of the UK, suggesting that they were all exposed in varying degrees to the British cultural norm of individualism. To more sharply gauge the influence of collectivistic cultural values on relationship quality and mate choice, in Study 2 we collected data from India and the United States. In India, one of the most collectivistic countries in the world [55, 59], parental influence on mate choice is high and arranged marriage still prevalent [66]. Alternatively, in the United States—a Western-individualistic country [59]—mate decisions are largely left up to individual preferences and parental influence is minimal [67].

A second improvement of Study 2 is that we replaced the Preferred Mate Attributes Scale [55] with the Ideal Partner Scale [68], allowing for a more refined measure of parent-child discrepancy in mate preferences. The Ideal Partner Scale is comprised of three factors that characterize mate preferences: warmth-trustworthiness, status-resources, and vitality-attractiveness. As such, we were able to gauge whether parent-child discrepancy in mate choice differed for each these three factors. Finally, Study 2 improved on Study 1 by recruiting a larger sample of participants who were currently involved in a relationship.

The hypotheses for commitment and passion were the same in Study 2 as in Study 1 (Hypotheses 1 and 2), but our hypothesis for parent-child discrepancy in mate choice was updated to reflect the factors of the Ideal Partner Scale. First, we predicted that collectivists’ higher family allocentrism would contribute to a smaller gap between their own choices for a mate with qualities signifying warmth-trustworthiness versus their perceptions of their parents’ choices (Hypothesis 3). Insofar as collectivist families are characterized by strong, interdependent ties, it is logical to surmise that both children and parents would agree that warm, trustworthy mates are most desirable, as these traits are likely to reinforce the family unit. We further postulated that collectivists’ higher parental influence would contribute to a smaller gap between their choices for a mate with qualities signifying status-resources versus their perceptions of their parents’ choices (Hypothesis 4). In collectivistic cultures, family reputation within the community and sharing of resources among family members is particularly prevalent. Insofar as parents benefit when their children marry into a family with economic success and strong social standing [49], they may pressure their children to marry partners of higher status. Finally, we did not make any predictions regarding mate characteristics denoting vitality-attractiveness. Across cultures, there are mate characteristics that both parents and children endorse because of their importance to both parties. Mates high in attractiveness-vitality signal health and fertility; insofar as these mates are more likely to produce healthy offspring, the genetic fitness of children and parents alike would benefit [14]. As such, we did not think that collectivism would be associated with discrepancies in parent-child preferences for attractive mates.

### Method

#### Participants

Three hundred and forty-six participants (160 women and 186 men; *M*_age:_ 29.35, *SD*: 9.24) were recruited for this study through Amazon’s Mechanical Turk. They were paid $0.35 (USD) for completion of the online survey. Of the total sample, 30% of participants indicated they were single and 70% stated they were currently involved in a relationship (dating, cohabitating, engaged, or married). The demographic statistics for this study is presented in Table 3.

**Table 3:** Demographic Statistics for Study 2.

Demographic Variables	Single Participants	Participants in a Relationship
	%	%
GENDER:		
Male	62	50
Female	38	50
AGE:	*M* = 25.38	*M* = 31.02
	*SD* = 6.44	*SD* = 9.73
*PARENTS KNOW ABOUT RELATIONSHIP:		*M* = 3.93*SD* = 1.37
COUNTRY OF RESIDENCE:		
India	49	44
United States	51	56
ETHNICITY OF INDIANS:		
White/Caucasian	0	1
Afro-Caribbean	0	1
South Asian	74	85
East Asian	14	7
Mixed	2	0
Other	10	6
ETHNICITY OF AMERICANS:		
White/Caucasian	79	83
Afro-Caribbean	6	2
South Asian	0	1
East Asian	6	9
Mixed	6	2
Other	3	3
PARTNER’S ETHNICITY:		Indian American
White/Caucasian		2 47
Afro-Caribbean		1 2
South Asian		81 37
East Asian		10 7
Mixed		0 1
Other		6 6

*Responses were measured on a 5-point Likert scale (1 = *My parents do not know I am in a relationship*, 3 = *My parents know I am in a relationship*, *but do not know many details*, 5 = *My parents have full knowledge that I am in a romantic relationship*).

#### Materials

Study 2 employed the same measures as Study 1, apart from the Ideal Partner Scale [68] and the continuous rather than categorical measure of dating experience. Cronbach’s alpha coefficients for the other scales were as follows: Parental Influence on Mate Choice (.90), Family Allocentrism (.91), Commitment (.87), and Passion (.95). Collectivism was operationalized in terms of cultural background, with participants from India classified as high in collectivism (1) and participants from the United States classified as low in collectivism (-1). In addition, the Horizontal/Vertical Individualism/Collectivism Scale [60] was once again administered to measure Americans and Indians’ level of collectivism. Cronbach’s alpha was. 82.

#### Ideal Partner Scale

Eighteen items from the short version of the Ideal Partner Scale [68] measure preferences for various mate attributes. The scale is comprised of three subscales: warmth-trustworthiness (e.g., “supportive”, “good listener”), vitality-attractiveness (e.g., “nice body”, “sexy”), and status-resources (e.g., “good job”, “financially secure”). We added four additional items based on measures by Buss and colleagues [55] and Lalonde and colleagues [24] because of their potential significance for choosing a mate in traditional, collectivistic societies (i.e., “comes from a family with a good reputation”, “favorable social status or rating”, “similar religious background” and “someone my family approves of”). Analogous to Study 1, participants rated the importance of each attribute for a potential marital partner, and how important it would be to their parents for their potential marital partner to possess these characteristics. Principal components analysis of this scale revealed that Fletcher et al.’s [68] three-factor structure cleanly emerged across groups for both children’s and parents’ marital mate preferences. The additional four items fully loaded on the status-resources factor. The three factors together accounted for 65% of the total variance in participants’ own mate preferences, and 64% of the variance in perceptions of parents’ mate preferences. Cronbach’s alphas for participants’ own mate preferences were as follows: warmth-loyalty (.90), vitality-attractiveness (.88), and status-resources (.93). Cronbach’s alphas for perceptions of parental mate preferences were as follows: warmth-trustworthiness (.91), vitality-attractiveness (.90), and status-resources (.90). Parent-child discrepancy was calculated as the absolute difference between the participant’s mate preferences and their perception of their parent’s mate preferences for each of the three subscales. Preferences were rated on a 7-point Likert scale ranging from 1 (*Very Unimportant*) to 7 (*Very Important*).

### Results and Discussion

Means, standard deviations, and Pearson’s correlations are reported in Table 4. Control variables included sex (-1 = female, 1 = male), age, and dating experience. Similar to Study 1, we included parents’ knowledge of their children’s relationship as a control variable in our analysis, but again our results were not influenced by this variable, and so it was removed from the following models. Supplementary analyses conducted on the variable assessing dating experience revealed that Indians who had more dating experience were more open with their parents about their dating life compared to Indians who had little dating experience, *r* = .49, *p* <. 0001. The association of dating experience with individual-level collectivism was not quite significant, *r* = -.14, *p* >. 09.

**Table 4:** Pearson’s correlations and descriptive statistics for Study 2.

Variable	1	2	3	4	5	6	7	8	9	10	11	Mean	*SD*
1. Sex	1.00											-	-
2. Age	-.06	1.00										29.35	9.24
3. Regularly Date	.08	.06	1.00									2.95	1.36
4. Collectivism	.12*	-.03	-.30**	1.00								-	-
5. Family Allocentrism	.08	-.11↑	-.26**	.54**	1.00							68.99	14.26
6. Parental Influence	.15**	-.05	-.28**	.78**	.64**	1.00						25.09	9.51
7. Commitment	-.20**	.08	-.20**	-.09	.16*	-.14*	1.00					29.57	5.70
8. Passion	-.04	-.01	-.18**	.23**	.30**	.20**	.65**	1.00				83.70	15.91
9. Parent-Child Discrepancy in Mate Preferences for Warmth-Loyalty	-.02	-.08	.06	-.08	-.21**	-.14*	-.07	-.12	1.00			3.95	5.12
10. Parent-Child Discrepancy in Mate Preferences for Vitality-Attractiveness	-.08	.05	.08	-.27**	-.17**	-.28**	.01	-.10	.36**	1.00		5.62	5.10
11. Parent-Child Discrepancy in Mate Preferences for Status-Resources	-.06	.13*	.07	-.36* **	-.13*	-.43**	.21**	-.01	.24**	.27**	1.00	10.88	10.66

↑ *p* <. 10. *p <. 05. **p <.01.

In addition, we included an effect-coded variable reflecting relationship status (1 = married, -1 = not married) and interactions of this variable with cultural group, parental involvement, and family allocentrism. None of the main effects of relationship status or interactions were significant except for the interaction of cultural group with relationship status on parent-child discrepancy in preferences for mates with status-resources indicating that married Americans had a larger discrepancy between their own and their parents’ preferences for mates with status-resources compared to unmarried Americans (*M*s = 18.55 and 12.30, *SD*s = 11.96 and 11.10, respectively), *t*(258) = -3.07, *p* <. 002). Given the lack of significant findings for main effects or interactions relevant to our mediational models, we removed these terms from our successive analyses.

We also repeated the *t*-test analysis that was conducted in the previous study to ascertain whether Indian participants (categorized as high in collectivism) were more collectivistic than American participants (categorized as low in collectivism). The results of the analysis confirmed our operationalization of culture: Indians (*M* = 31.96, *SE* = 4.35) scored higher in collectivism than Americans (*M* = 25.36, *SE* = 4.35), *t*(323) = -13.24 *p* <. 0001. As in Study 1, Preacher and Hayes’s [63] bootstrap method was used to examine the indirect effects of collectivism on commitment, passion, and parent-child discrepancy through parental influence and family allocentrism.

According to Hypothesis 1, collectivism should exert a negative indirect effect on commitment through parental influence, but a positive indirect effect through family allocentrism. Fig. 3 shows that the total effect of collectivism on relationship commitment was significant (*b* = -.84, *p* <. 03), whereas the direct effect of collectivism (i.e., controlling for the indirect effects of parental influence and family allocentrism) was not significant (*b* = -.23, *p* >. 10). Confirming Hypothesis 1, the indirect effect of collectivism through parental influence was significant and negative [*b* = -1.67 (CI: -2.66, -.80)], whereas the indirect effect of family allocentrism was significant and positive [*b* = 1.06 (CI:. 61, 1.77)].

Hypothesis 2 asserted that collectivism would exert a negative indirect effect on passion through parental influence and a positive indirect effect through family allocentrism. As indicated in Fig. 4, the total effect of collectivism on passion was significant (*b* = 2.78, *p* <. 02), but the direct effect of collectivism was not (*b* = 1.81, *p* >. 10). Partly confirming Hypothesis 2, the indirect effect of collectivism on passion through family allocentrism was positive and significant [*b* = 2.12 (CI:. 64, 4.53)], whereas the indirect effect of parental influence was not.

**Fig 6:**
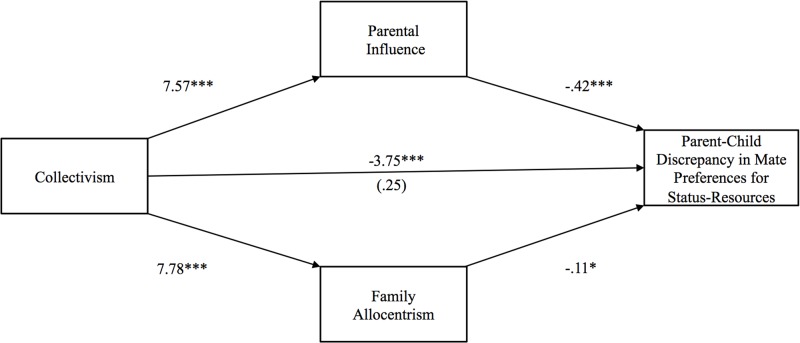
Study 2: Indirect effect of collectivism on parent-child discrepancy in mate selection for qualities signifying status-resources through parental influence and family allocentrism. The value in parentheses represents the direct effect, and the value directly above is the total effect. * p <. 05, ** p <. 01, ***p <. 001.

Hypothesis 3 postulated that collectivism would exert a negative indirect effect on parent-child discrepancy in preferences for mates with qualities denoting warmth-trustworthiness through family allocentrism. As shown in Fig. 5, this indirect effect was significant [*b* = -.61 (CI: -1.41, -.03)], but the indirect effect through parental influence was not. Confirming Hypothesis 4, the indirect effect of collectivism on parent-child discrepancy in preferences for mates with status-resources through parental influence was significant [*b* = -3.12 (CI: -4.87, -1.64)], but the indirect effect through family allocentrism was not (see Fig. 6). Finally, the indirect effects of collectivism on parent-child discrepancy in preferences for mates with qualities representing vitality-attractiveness through parental influence and family allocentrism were not significant.

It is important to note that analyses were also performed which excluded US participants who reported an ethnic heritage that was coded as collectivist in Study 1 (i.e., South and East Asians). A total of 19 participants were omitted; the remaining 327 participants identified as 90% White/Caucasian, 4% as African/ Caribbean, 3% mixed, and 3% other. The results of the analyses indicated that all our associations were still in the same direction with similar *p*-values in accordance with our original results when these participants were removed. The only difference was for the model predicting the dependent variable parent-child discrepancy in preferences for mates with qualities designating warmth-trustworthiness. When South and East Asians were removed from the analysis, family allocentrism became a slightly weaker predictor of the dependent variable given the smaller sample size—the *p*-value in the first analysis was. 004; in the second analysis, it was. 01. Therefore, we postulate that the slightly weaker predictive power of family allocentrism, while still significant, may be responsible for the non-significant indirect effect when South and East Asians participants were removed from the study.

Consistent with the findings of Study 1, then, these results showed that the direct effect of collectivism on commitment became more positive after accounting for the downward pressure from parental influence and the upward pressure from family allocentrism. In both studies, the indirect effect of collectivism on commitment through parental influence was negative, while the indirect effect through family allocentrism was positive; only in Study 2, though, we were able to show that the indirect effect through family allocentrism was significant. When family allocentrism is high, family members may feel a sense of closeness and devotion to each other that may allow them to express a similar sense of commitment toward a romantic partner. This sense of closeness may also help to explain why collectivists reported greater passion in their relationship. Nevertheless, as individuals from collectivistic cultures remain dependent on family approval for their mate selection [31], parental influence may suppress the positive effects of family allocentrism on commitment. Finally, Study 2 found that the smaller gap between collectivists’ own preferences for partners demonstrating warmth and trustworthiness and their perceptions of their parents’ preferences for these qualities was explained by the closeness and connection shared between family members. Alternatively, the similarity between collectivists’ own preferences for a partner with status and resources and their perceptions of their parents’ preferences for these qualities was explained by their parents’ greater involvement in their love life.

## General Discussion

The purpose of this research was to test whether collectivism predicted relationship commitment, passion, and parent-child discrepancies in mate preferences because of cultural emphases on parental authority and family allocentrism. In Study 1, we found that individuals from collectivistic backgrounds accepted higher parental influence on their mate choice, which exerted downward pressure on their level of commitment in a relationship. Study 2 confirmed these findings and further showed that collectivists’ greater family allocentrism was likely to apply upward pressure on their commitment. Furthermore, the results of Study 1 suggested that parental influence inhibited collectivists’ passion, whereas Study 2 suggested that family allocentrism may enhance it. While we were unable to show that parental influence and family allocentrism applied opposing pressures on passion within a single study, the results of Studies 1 and 2 separately suggested that family allocentrism drives up collectivists’ passion, whereas parental influence damps it down. Taken together, the results of both studies demonstrate the prospective latent struggles that collectivists may experience in their romantic relationships as they try to manage these opposing forces. Furthermore, Study 2 revealed that collectivists’ tendency to experience higher family allocentrism contributed to the smaller discrepancy between their own preferences for a mate with qualities signifying warmth-trustworthiness and their perception of their parents’ preferences; meanwhile, higher parental influence contributed to a smaller gap between their own preferences for a mate with qualities signifying status-resources and their perception of their parents’ preferences. We discuss these results in greater detail below.

Insofar as collectivists have a strong sense of duty to in-groups and cultivate interdependent ties [2], it is logical to presume that they would highly value commitment to romantic partners. Indeed, Luo [29] found that second-generation Chinese-American youth negatively perceived American causal dating behaviors, preferring committed relationships instead. In addition, collectivists’ strong sense of responsibility and connection to family members often means that selecting a marital partner involves parental input, with both children and parents working together to choose a suitable mate [69]. This greater acceptance of parental involvement in children’s mate selection process [3] may sway commitment towards a romantic partner based upon the approval or disapproval of parents. Studies 1 and 2 both found that participants from collectivistic backgrounds reported greater parental influence on their mate selection process and, in turn, reported lower levels of commitment. These results are consistent with the findings of MacDonald and colleagues [31], who found that collectivists facing parental disapproval were less likely to invest in their relationship.

Alternatively, in Study 2, we found that participants from India, a highly collectivist country, were higher in family allocentrism than Americans; in turn, Indians reported greater relationship commitment, suggesting that family allocentrism and parental influence contributed to commitment in opposite ways. While family allocentrism also encourages involvement of parents in their children’s romantic relationships, children may perceive this involvement differently than in the case of direct parental influence. Whereas parental influence highlights the authority parents can have over their children’s choices, family allocentrism emphasizes the mutual devotion and attachment family members feel toward one another [3, 5]. Consequently, if individuals experience a higher degree of family allocentrism with their immediate family members, it may be that they desire a similar degree of allocentrism with their romantic partner, contributing to an elevated degree of commitment in their relationship. These findings may help to explain discrepancies in the current literature on romantic relationships within collectivistic cultures [29, 70, 71]: individuals may experience conflicting pressures on their romantic commitment as a result of inconsistent familial and cultural messages.

Another facet of relationship quality we examined was passion. Consistent with research that has found a negative link between parental influence and romantic beliefs [3], in Study 1 we found that parental influence was negatively associated with passion, a correlate of romantic beliefs. Insofar as one’s family practices greater authority over one’s love life, one may attribute less importance to such relationship factors as chemistry or passion. Instead one may deem extra-dyadic factors—such as nurturing a positive bond between one’s own and one’s partner’s family members—to be more vital, mitigating the need for passion within their own romantic relationship [72]. On the other hand, Study 2 revealed that young adults from a more collectivistic culture reported greater family allocentrism and, in turn, reported stronger passion in their relationship. If these individuals already sustain strong family relationships, it may be that the bond with their family members has encouraged a desire for a similar connection in romantic relationships, cultivating a stronger sense of passion within their relationship.

Finally, we examined the predictors of preferences for a marital partner. Past research in cross-cultural psychology has explored the desirability of various mate attributes principally within a collectivistic-individualistic context [24]. Our aim was to extend this research by examining whether parental influence and family allocentrism mediated the association of collectivism with discrepancies between parents’ and children’s preferences for mates. While Study 1 did not demonstrate any significant mediation, we believe this stemmed from two factors. First, principal components analysis of the measure of mate preferences utilized in Study 1 yielded only one dominant factor, allowing for a less refined measure of preferred mate characteristics. Additionally, while the group classified as highly collectivistic in Study 1 were from heritage cultures identified by Hofstede [59] as high in collectivism, these participants were also living in the United Kingdom—an individualistic society—and may not have experienced the full weight of having to comply with collectivist values and expectations, as did their counterparts in Study 2.

On the other hand, the results in Study 2 indicated that participants from collectivistic backgrounds reported greater family allocentrism and, in turn, reported a smaller gap between their marital mate preferences and their perceptions of their parents’ preferences for a mate with qualities signifying warmth and trustworthiness. Family allocentrism generates sentiments of warmth and loyalty between family members; as a result, socialization within such a close family unit may mean that individuals seek equal virtues in a marital partner.

Furthermore, Study 2 revealed that collectivists reported greater parental influence on their mate choice and, in turn, reported a smaller gap between their marital mate preferences and their perceptions of their parents’ preferences for a mate with qualities characterizing status-resources. Marriage is a public act, with children’s choices in a mate frequently reflecting the reputation of the entire family, especially in collectivistic countries [55]. Consequently, inasmuch as a child’s spouse becomes a part of the family unit, it is often important to parents for their children to select a mate who can contribute to the overall well-being and prosperity of the entire family. When parental influence is high, it appears that children may internalize these beliefs, showing a similar interest in mate qualities that denote status and resources in line with their parents’ wishes [15].

As for traits indicating vitality-attractiveness, no significant cultural difference was found in the discrepancy between children and parents’ preferences. Evolutionary theory suggests that healthy offspring is the key to genetic fitness [73]. Across cultures, then, both parents and young adults may overlap in their desire to select a mate with traits that connote vitality in an attempt to maximize the health of their children [14, 55].

Although we were unable to demonstrate in both studies that parental influence and family allocentrism simultaneously exerted indirect effects on the associations of collectivism with all of our dependent variables, we believe this was chiefly due to discrepancies in data collection. As mentioned above, many of the participants in Study 1 were second-generation immigrants living in the UK, whose parents originated from more collectivistic countries. Living among their Western peers, who most likely enjoyed free-choice in their romantic relationships, the participants classified as highly collectivistic in Study 1 may have perceived their own level of parental influence on their romantic relationship outcomes more heavily. In an attempt to gain more freedom and autonomy, like their Western friends, they may have pushed against and sought more distance from their family members, reducing family allocentrism relative to their collectivist counterparts in Study 2. The sharper cultural difference in family allocentrism in Study 2 may explain why the indirect effect of collectivism on relationship commitment and passion through family allocentrism was significant here but not in Study 1.

### Limitations and Future Directions

Although our findings have offered important insights into cultural influences on relationship quality and partner selection, they also have certain limitations. Study 1 focused on the influence of collectivistic cultural values among first- and second-generation immigrants in the United Kingdom, but did not take into account the role of acculturation beyond assessing generational status. Future research would do well to assess the influence of acculturation strategies [74] on migrants’ perceptions of parental influence on mate choice, family allocentrism, relationship quality, and mate preferences. For example, migrants with assimilationist tendencies who adopt Western-style attitudes towards parental influence on mate choice may report greater commitment and passion in premarital relationships; however, such enhancements may be offset to the extent that they also experience a reduction in family allocentrism.

It is important to note that the results of Study 2 may not be generalized beyond our particular sample of Indian participants who may have come from predominately middle or upper class backgrounds and potentially experienced increased exposure to individualistic concepts and norms. These individuals spoke English, owned computers, and were more open to conventionally individualistic customs such as dating; that there existed a positive association of dating experience with openness with parents is suggestive of these Indians’ more individualistic inclinations [17]. However, Indians may be simultaneously high in collectivism and individualism, endorsing each value system depending on context [75]. Nevertheless, given the ample differences in religion, language, cast, and socioeconomic status one can find in India, our sample may not be indicative of the mainstream population of Indian youth. Additional insight might also be gained by sampling participants from a wider selection of cultures that vary in collectivism.

Another shortcoming of this research is that we asked participants to rate their *perceptions* of their parents’ marital preferences for their children rather than obtain ratings from the parents themselves. However, one could argue that it is the participants’ perception of their parents’ preferences that may be most predictive of the dependent variables. Studies have shown that there are frequently incongruities between parents’ reported personal values and their socialization values—the values that parents ultimately transmit to their children [76, 77]. Indeed, a number of researchers have shown that it is children’s *perception* of parents’ beliefs and not necessarily the parents’ *actual* beliefs—acquired through parental self-reports—that are most predictive of children’s own value formation in the family [78, 79, 80, 81]. Parent-child value transmission has been conceptualized as a two-pronged process; the first noteworthy step is children’s perception of parental values and the second is their willingness to accept parental messages [54]. Nevertheless, it would be worthwhile for future research to collect data directly from parents to more accurately gauge parent-child discrepancies in mate choice.

Additionally, further studies should examine parental influence not only from one’s own family of origin, but also from one’s *partner’s* family. A person’s investment in a relationship may waver, not only because of their own family’s disapproval, but also because of their partner’s family’s disapproval [31]. Finally, the positive association between family allocentrism and passion in our study was somewhat tenuous. Further replication should be done, especially after controlling for intimacy, to gain additional insight into this somewhat surprising finding.

### Concluding Remarks

Our research sought to disentangle the influence of collectivism on relationship quality and mate preferences by examining the mediating roles of parental influence and family allocentrism. Two studies showed that collectivists experienced upward pressure on their relationship commitment and passion due to their family allocentrism, but they experienced concurrent downward pressure on these relationship outcomes due to high parental influence, potentially creating ambivalence. We further found that collectivists’ tendencies to experience higher family allocentrism explained their smaller discrepancy in their preferences and their perception of their parents’ preferences for a mate possessing qualities of warmth and trustworthiness, while their higher acceptance of parental influence narrowed the gap in parent-child preferences for mates with status and resources. Further research examining the influence of conflicting cultural ideologies on mate preferences and relationship quality may assist practitioners in helping people to resolve personal ambivalence and intergenerational tension.
